# The epidemiology of MRI detected shoulder injuries in athletes participating in the Rio de Janeiro 2016 Summer Olympics

**DOI:** 10.1186/s12891-018-2224-2

**Published:** 2018-08-17

**Authors:** Akira M. Murakami, Andrew J. Kompel, Lars Engebretsen, Xinning Li, Bruce B. Forster, Michel D. Crema, Daichi Hayashi, Mohamed Jarraya, Frank W. Roemer, Ali Guermazi

**Affiliations:** 1 Section of Musculoskeletal Imaging, Department of Radiology, Boston University School of Medicine, FGH Building, 3rd Floor, 820 Harrison Ave., Boston, MA 02118 USA; 2 Medical and Scientific Department, International Olympic Committee, Lausanne, Switzerland; 3 Oslo Sports Trauma Research Center, Department of Sports Medicine, Norwegian School of Sport Sciences, Oslo, Norway; 4 Department of Orthopedic Surgery, Oslo University Hospital, University of Oslo, Oslo, Norway; 5 Department of Orthopaedic Surgery, Boston University School of Medicine, Boston, MA USA; 6 Department of Radiology, University of British Columbia, Vancouver, BC Canada; 7 Department of Sports Medicine, National Institute of Sports (INSEP), Paris, France; 8 Department of Radiology, Saint-Antoine Hospital, University Paris VI, Paris, France; 9 Department of Radiology, Stony Brook Medicine, Stony Brook, NY USA; 10 Department of Radiology, Mercy Catholic Medical Center, Darby, PA USA; 11 Department of Radiology, University of Erlangen-Nuremberg, Erlangen, Germany

**Keywords:** MRI, Olympics, Shoulder, Injury

## Abstract

**Background:**

To use Magnetic Resonance Imaging (MRI) to characterize the severity, location, prevalence, and demographics of shoulder injuries in athletes at the Rio de Janeiro 2016 Summer Olympic Games.

**Methods:**

This was a retrospective analysis of all routine shoulder MRIs obtained from the Olympic Village Polyclinic during the Rio 2016 Summer Olympics. Imaging was performed on 1.5 T and 3 T MRI, and interpretation was centrally performed by a board-certified musculoskeletal radiologist. Images were assessed for tendon, muscle, bone, bursal, joint capsule, labral, and chondral abnormality.

**Results:**

A total of 11,274 athletes participated in the Games, of which 55 (5%) were referred for a routine shoulder MRI. Fifty-three (96%) had at least two abnormal findings. Seven (13%) had evidence of an acute or chronic anterior shoulder dislocation. Forty-nine (89%) had a rotator cuff partial tear and / or tendinosis. Subacromial / subdeltoid bursitis was present in 29 (40%). Thirty (55%) had a tear of the superior labrum anterior posterior (SLAP).

**Conclusion:**

Our study demonstrated a high prevalence of both acute and chronic shoulder injuries in the Olympic athletes receiving shoulder MRI. The high rates of bursal, rotator cuff, and labral pathology found in these patients implies that some degree of glenohumeral instability and impingement is occurring, likely due to fatigue and overuse of the dynamic stabilizers. Future studies are needed to better evaluate sport-specific trends of injury.

## Background

The 2016 Rio de Janeiro Summer Olympic Games were held from August 5 to 21, 2016, bringing together 11,274 elite athletes from 206 different countries and a team of refugees. In this elite international competition, 8% of athletes incurred at least one injury during participation. Forty percent of these injuries resulted in loss of competition for at least 1 day, and 20% of the injuries resulted in loss of competition for greater than 7 days [1, 2].

Shoulder injuries constitute a small subset of all injuries at the Olympic Games, however the pain associated with even chronic conditions such as tendinosis can result in significant pain symptoms [3]. The relative lack of osseous restraint within the glenohumeral joint allows for the mobility and flexibility required for high level athletic performance. However, it also places a high physical demand on the static and dynamic stabilizers of the shoulder, particularly in athletes at this level. Overuse injuries to the rotator cuff and shoulder girdle muscles as well as to the labrum, joint capsule, and cartilage can result in instability and impingement of the joint, impeding performance [4].

Shoulder pain and injury has been particularly well documented in sports with repetitive overhead motions [3, 5–8]. While the overhead throwing athlete in particular seems most at risk, there are many Olympic sports in which similar demands are placed on the glenohumeral joint. High level athletes in contact sports such as rugby and American football can also sustain similar injuries even when overhead throwing motions are not inherent in their particular activity [9–11]. While various imaging modalities have been used for diagnosis, magnetic resonance imaging (MRI) has been the established imaging modality of choice in evaluating such conditions [4].

The aim of our study is to use MRI to characterize the severity, location, prevalence, and demographics of acute and chronic shoulder injuries observed at the Rio de Janeiro 2016 Summer Olympic Games, in order to better anticipate athlete diagnosis and care in future events of an elite caliber.

## Methods

This is a retrospective analysis of the patient information from the International Olympic Commission (IOC) athlete database and imaging data from the Radiological Information System (RIS) and Picture Archiving Communications System (PACS) of the Rio 2016 Summer Olympics. The earliest imaging was performed 6 days prior to the opening ceremonies, and the last study was acquired 1 day after the closing ceremony. The assigned athlete accreditation number was used to query the IOC database for demographic information, which included age, gender, nationality, and sport. All information was treated with strict confidentiality, and our medical database was de-identified. The study was approved by the medical research ethics committee of the South-Eastern Norway Regional Health Authority (2011/388) and was exempt from Ethics Committee approval. Informed written consent was waived since all epidemiological data was anonymized and unidentifiable. The use of anonymized imaging and demographic data for publication was approved by the IOC. An additional Institution Review Board (IRB) was obtained from Boston University (#H-36593). The data was collected, stored, and analyzed with strict compliance to data protection and athlete confidentiality.

All patients were imaged at the official IOC polyclinic in the Olympic Village using either a 3 T Discovery MR750w or 1.5 T Optima 450MRw MRI scanner (General Electric, Waukesha, Wisc). MRI sequences consisted of 3 planes (axial, oblique coronal, oblique sagittal) of fluid sensitive T2-weighted or proton density (PD)-weighted fat-suppressed sequences. A coronal or sagittal T1-weighted sequence was also acquired. Neither intravenous nor arthrographic Gadolinium was utilized.

### Image interpretation

A board certified, subspecialty radiologist (AM) with 8 years of musculoskeletal imaging experience including imaging of sports injuries, retrospectively reviewed all MRI examinations. The radiologist was blinded to the official imaging report. All data was recorded on a Microsoft Excel spreadsheet, and a descriptive statistical analysis was performed.

Osseous lesions were characterized based on location and bone marrow signal characteristics. Any bone marrow hyperintensity or edema pattern on either a T2 or PD-weighted fat-suppressed sequence was considered a bone contusion. Any bone marrow edema pattern associated with osseous fragmentation or a low linear signal on the T1-weighted sequence was considered an acute fracture [12]. Hill Sachs and osseous Bankart lesions were diagnosed based on their characteristic locations on the posterior superior humeral head and anterior inferior glenoid, respectively. A designation of either acute or chronic was based on the presence of a bone marrow edema pattern [13, 14].

Fluid within the subacromial / subdeltoid bursa and within the glenohumeral joint was assessed on the T2 or PD-weighted fat-suppressed sequences. Any region of hyper-intense subacromial or subdeltoid bursal thickening that was 2 mm or greater was considered a bursitis [4]. A glenohumeral joint effusion was characterized on a 4-point scale as reported by Schweitzer et al. Normal intra-articular fluid produces a thin intraarticular rim of hyperintense signal, but without distension of a joint recess. The presence of slight fluid distension of the subscapular recess, fluid within the biceps tendon sheath, or fluid within the axillary recess was considered a small glenohumeral joint effusion. Fluid distension of two of these recesses represented a moderate sized effusion. Fluid distension of all three structures represented a large effusion [15].

Each rotator cuff tendon was evaluated for the presence of tendinosis or tear. Tendinosis was diagnosed by the presence of signal hyperintensity on the PD and to a lesser extent T2-weighted images, and or in the presence of tendon thickening without fiber discontinuity [16, 17]. Any morphologic defect of the tendon fibers, either along the bursal surface, intrasubstance, or articular surface that was filled with fluid signal, particularly on the T2-weighted sequences, was considered a partial thickness defect. A full thickness tear was considered present if the morphologic defect in the tendon fibers extended from articular surface to bursal surface [18].

Any muscle injury of the deltoid or rotator cuff was characterized using an MRI-modified version of the Peetrons classification system [19, 20]; grade 1 – ill-defined hyperintensity on the fluid sensitive sequences indicating edema signal without architectural distortion of muscle fibers or macroscopic tear; grade 2 – architectural distortion of muscle fibers or well-defined hyperintensity on fluid sensitive sequences indicating partial muscle tear; and grade 3 – total muscle tear with retraction.

The severity of rotator cuff muscle atrophy was assessed using the Goutallier classification system, which has been validated for use in both CT and MRI [21, 22]; grade 1 – some fatty streaks; grade 2 – fatty infiltration, but more muscle than fat; grade 3 – moderate fatty infiltration but as much fat as muscle; and grade 4 – severe fatty infiltration with more fat than muscle.

The morphologic contour and signal of the labrum was assessed for the presence or absence of tears on the T2 or PD-weighted sequences with or without fat suppression. Diagnostic criteria for a tear included the presence of intrasubstance labral high signal, irregular labral margins, high intrasubstance signal that was non-parallel to the glenoid margin, high signal intensity either posterior to the long head of the biceps origin or inferior to the three o’clock position, or a separation of glenoid and labrum that was greater than 2 mm. A special distinction of labral tear in association with a Bankart lesion was made [23, 24].

The presence of joint capsule abnormality of the acromioclavicular (AC) joint or the anterior inferior glenohumeral ligament of the glenohumeral joint was evaluated. An acute capsular injury was diagnosed by the presence of either a frank capsular defect, or by the abnormal morphology and edema signal both within the capsule and along the extracapsular margin. A chronic capsular injury or abnormality was diagnosed by the observation of a scar thickened joint capsule or capsular defect but with an absence of extracapsular edema signal [23, 25].

Injuries of the long head of the biceps were characterized as involving either the extra-articular vertical portion or the intra-articular horizontal portion. The severity of injury was assessed by the presence of either tendinosis, partial tear, or rupture. Tendinosis was defined as biceps tendon thickening and /or high intrasubstance signal on the fluid sensitive sequences. A partial thickness tear was defined as any focal tendon caliber change or high intrasubstance signal approaching fluid intensity. A complete tear was defined as a complete discontinuity of the tendon [26].

Lastly, cartilage defects were evaluated using a modified Outerbridge classification system on fluid sensitive sequences [27, 28]; grade 1 – heterogeneous signal; grade 2 – shallow ulceration, fibrillation, or fissuring < 50% depth; grade 3 – deep ulceration, fibrillation, fissuring or chondral flap > 50% depth; grade 4 – full thickness loss and exposed subchondral bone.

## Results

A total of 11,274 athletes which included 5089 women (45%) and 6185 men (55%) participated in the 2016 Olympic Games. The National Olympic Committees and Rio 2016 medical staff evaluated a total of 1101 acute and chronic injuries during the course of the games. Of these injured athletes, 55 (5%) were referred for MRI of the shoulder for further evaluation of shoulder pain and injury. The 55 patients included 28 males (51%) and 27 females (49%) with an average age of 26 years, ranging from 18 to 34. The patients came from 20 different Olympic sports; of these, swimming (6), judo (6), boxing (5), gymnastics (5), volleyball (5), and athletics (track and field) (4) provided the most number of patients. Of all the MRIs, only 2 (4%) were considered completely normal. The remaining 53 (96%) MRI studies each had two or more abnormal findings.

### Osseous abnormalities

Two patients presented with an acute fracture. This included an acute Hill Sachs deformity and one highly comminuted fracture of the scapula. Seven patients sustained a bone contusion by study criteria.

### Anterior instability

**Fig. 1:**
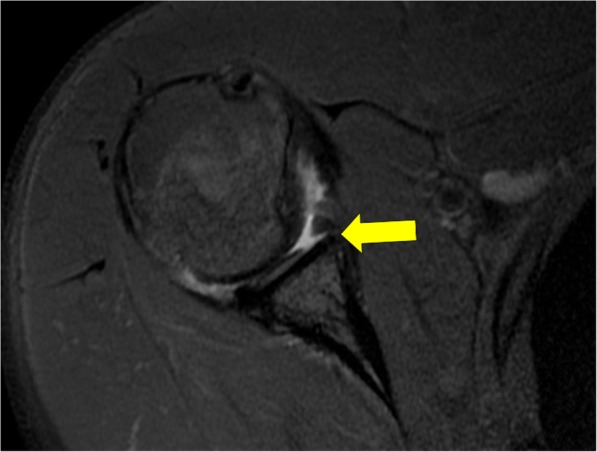
Female rugby player in her late 20’s: Axial T2-weighted fat-suppressed MRI demonstrates tear of the anterior inferior labrum (Bankart) lesion (arrow) and adjacent high grade chondral loss over the glenoid

Seven patients presented with evidence of an anterior shoulder dislocation. Boxing, taekwondo, rugby, athletics (track and field), judo, basketball, and wrestling were each represented. One of these patients sustained an acute Hill Sachs injury in combination with a soft tissue Bankart lesion (anterior inferior labral tear, Fig. 1) and acute capsular tear of the anterior inferior glenohumeral ligament. The six other patients had chronic Hill Sachs deformities as evidenced by the classic bony contour abnormality of the superior humeral head, but with a lack of bone marrow edema signal. Two of the patients also had an osseous Bankart deformity, while the remaining four patients had a purely soft tissue displaced anterior inferior labral tear.

### Rotator cuff

Abnormalities of the rotator cuff were common in this sample, being observed in up to 49 patients (89%). Swimming, volleyball, judo, gymnastics, and track and field provided the most patients. The highest proportion of athletes per number of participants came from volleyball, judo, and gymnastics. Distribution of rotator cuff injury are listed by sport (Table 1).

**Table 1:** Distribution of rotator cuff abnormality by per sport

Sport	Patients	Total Number of Participants	Fraction of patients to the # of participants
Aquatics - Swimming	6	901	0.007
Volleyball	5	288	0.017
Judo	5	390	0.013
Gymnastics - Artistic	5	194	0.026
Athletics (Track and Field)	4	2367	0.002
Wrestling	3	349	0.009
Cycling - Road	3	211	0.014
Boxing	3	289	0.010
Tennis	2	199	0.010
Rugby	2	291	0.007
Handball	2	335	0.006
Weightlifting	1	256	0.004
Taekwondo	1	127	0.008
Hockey	1	384	0.003
Football	1	503	0.002
Field Hockey	1	384	0.003
Canoe - Sprint	1	248	0.004
Beach volleyball	1	96	0.010
Basketball	1	287	0.003
Aquatics - Water polo	1	258	0.004

**Fig. 2:**
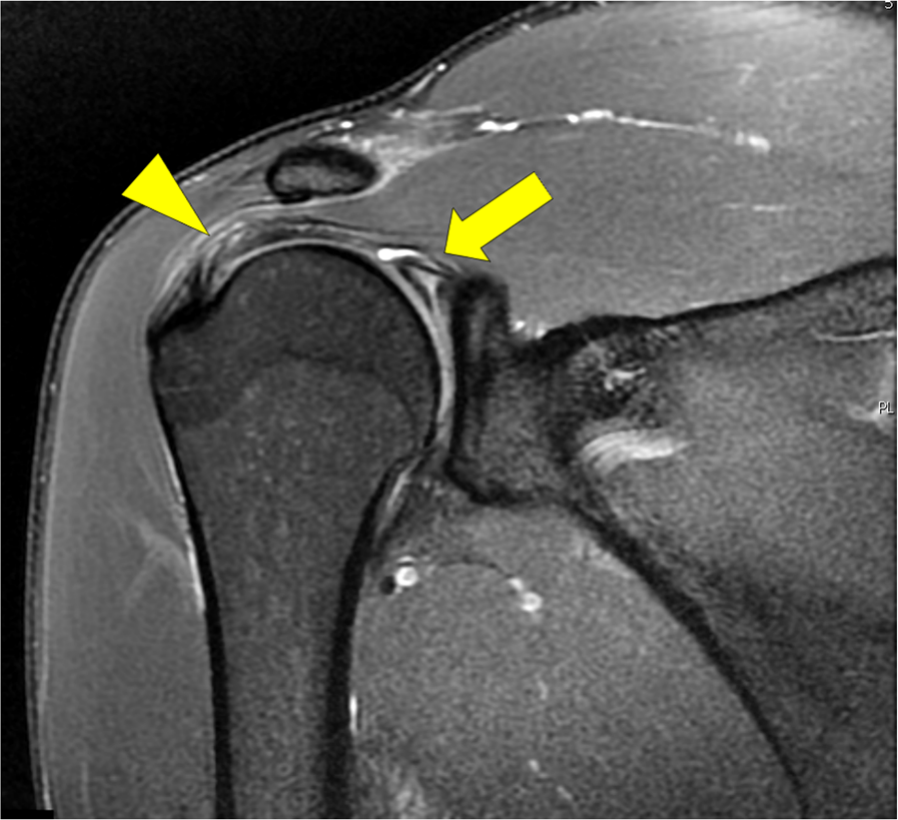
Male gymnast in his late 20’s: Coronal T2-weighted fat-suppressed MRI shows superior labral tear and overlying paralabral cyst (arrow) and a low grade intrasubstance tear of supraspinatus tendon (arrowhead)

**Fig. 3:**
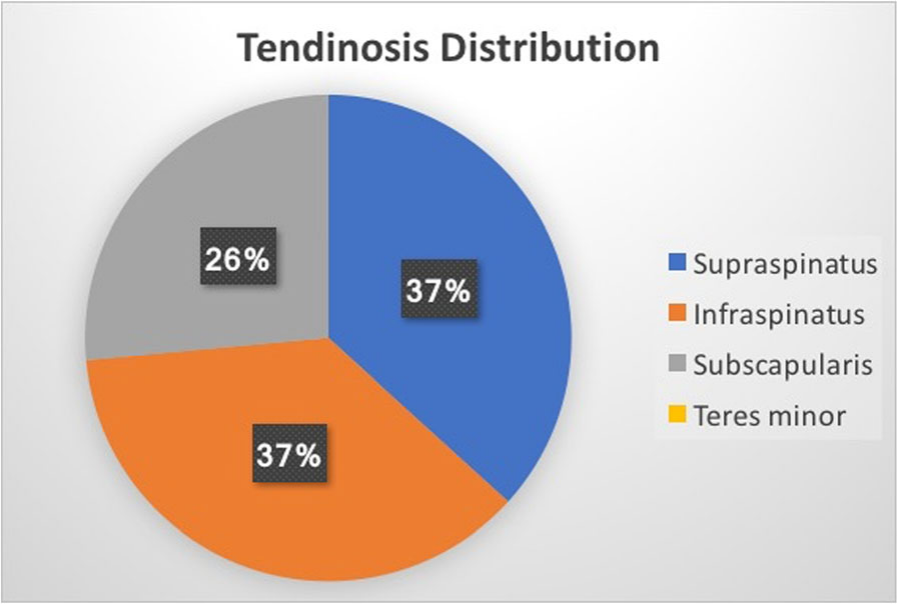
Distribution of rotator cuff tendinosis

**Fig. 4:**
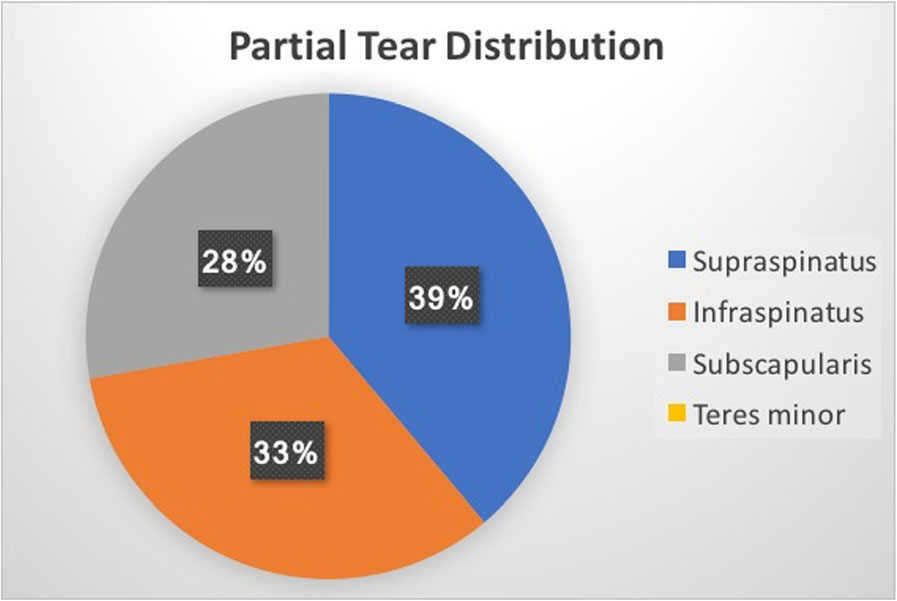
Distribution of rotator cuff partial tear

Of the total patients, 22 presented with tendinosis only, while 27 had a partial thickness tear of the tendon (Fig. 2). There were no patients with either full thickness or complete rupture of a tendon. Nine patients demonstrated additional edema signal within the muscle and myotendinous junction of the rotator cuff which was interpreted as an acute, low grade muscle strain. The distribution of the rotator cuff tendons involved in either tendinosis (Fig. 3) or partial tear (Fig. 4) was similar.

### Labrum

Tears of the superior labrum anterior posterior (SLAP), were relatively common, seen in 30 patients (55%). Thirteen of these SLAP tears were accompanied with abnormalities of the intra-articular long head of the biceps; seven patients had a partial thickness tear of the biceps with their SLAP tear, while 6 had at least tendinosis of the biceps. The distribution of SLAP tears are listed by sport (Table 2). Gymnastics had the highest proportion of SLAP tears relative to the total number of Olympic participants.

**Table 2:** Distribution of superior labrum anterior posterior (SLAP) tears by sport

	Cases	Total Number of IOC Participants	Fraction
Judo	4	390	0.010
Gymnastics	4	194	0.021
Wrestling	3	349	0.009
Volleyball	3	288	0.010
Athletics (Track and field)	3	2367	0.001
Swimming	3	901	0.003
Tennis	2	199	0.010
Rugby	2	291	0.007
Cycling - Road	2	211	0.009
Football	1	503	0.002
Boxing	1	289	0.003
Basketball	1	287	0.003
Water polo	1	258	0.004

### Long head of the biceps tendon

Abnormalities of the biceps tendon were present in 16 patients. Only 1 had involvement of the vertical portion, which presented as a partial tear. The remainder of these patients had involvement of the horizontal portion of the tendon. Thirteen of these patients (87%) were in association with a SLAP tear and are described in detail above. The remaining 2 patients had tendinosis.

### Bursa

A subacromial / subdeltoid bursitis was present in 29 patients (40%).

### Joints

A joint effusion was seen in 23 patients (42%). In all but three of these patients, the size was considered small.

Degenerative glenohumeral chondral loss was noted in 13 total patients (24%). Track and field (3) and gymnastics (2) provided the most patients. Of the total, 10 patients had chondral defects that were considered either an Outerbridge 3 or 4. Five of the patients (38%) with chondral loss had either a Hill Sachs deformity or osseous/soft tissue Bankart lesion or both. Nine of these patients (69%) with chondral loss also had a SLAP tear.

In regards to the AC joint, four patients demonstrated capsular defects, two acute and two chronic. Sixteen patients had evidence of either mild to moderate chondral loss of the AC joint.

## Discussion

Acute and chronic shoulder injuries are common problems in elite athletes. Sports with overhead throwing activities have been most frequently studied, with the prevalence of shoulder pain reported to be anywhere between 23 and 36% [7, 8]. This is ultimately attributed to the relatively unnatural and highly dynamic nature of the throwing movement. A strict balance of the dynamic and static stabilizers are needed to maintain a stable center of rotation [29], and loss or damage of these supporting structures can lead to shoulder pain as well as a decrease in performance. There are many additional sports in which repetitive overhead motion is inherent in competition, also leading to shoulder pain. For example, in elite, competitive swimmers, limiting shoulder pain has been reported to be between 40 and 90% [30–32]. In such cases, the etiology is most likely similar and backed by studies which have analyzed glenohumeral kinematics; With increasing rotator cuff fatigue, there is superior migration of the humeral head during arm elevations which leads to impingement and rotator cuff injury [33, 34].

Our findings are in line with this etiologic theory, given the high percentage of rotator cuff abnormalities and subdeltoid bursitis occurring in athletes involved in contact or overhead motion sports. In comparison to the general population, a review of the published literature performed by Teunis et al. [35] found that the prevalence of any rotator cuff abnormality (tendinosis or tear) is 9.7% in individuals less than 20 years, and 6.9% in those from 20 to 29 years. This is in contrast to our study patients, in which 89% demonstrated a rotator cuff abnormality (tendinosis or tear). The relatively even tendon distribution of the tears and tendinosis involving supraspinatus, infraspinatus, and subscapularis does slightly deviate from the previously described pathologic continuum of chronic subacromial impingement leading to predominantly supraspinatus tendinosis and tear [36]. However, the diversity of cuff involvement evident in these athletes may reflect the diversity of sports.

Labral injury, like rotator cuff injury, generally has a high prevalence in symptomatic and asymptomatic elite athletes. While the incidence is most commonly associated in sports with repetitive overhead motion or throwing, it has also been seen in contact athletes [4–6, 9]. For example, the incidence of SLAP tears in elite rugby players has been found to be as high as 83% [10, 11]. Our reported overall occurrence of SLAP tears at 55% is comparable to prior published studies on elite athletes. Also in keeping with the published data, the distribution of labral tears is mostly with sports with inherent overhead motion or contact.

Of patients with glenohumeral chondral loss, 85% had either evidence of anterior shoulder instability and prior dislocation and / or a superior labral tear. This is an entirely expected finding, as it is established that chronic shoulder instability can eventually lead to chondral loss. Previous studies have demonstrated that the incidence of chondral damage to be as high as 9.2% in patients with anterior shoulder instability [37] and up to 64% in patients undergoing arthroscopic Bankart repair [38].

Our study had several inherent limitations. As a strictly observational study, we did not analyze the medical record to correlate the clinical exam findings with the reason of study. Other than the clinical diagnosis of shoulder pain as an indication for MRI, we lacked detailed knowledge of clinical information that would better link our imaging findings to the clinical presentation. Our patient population inevitably included both acute injuries and pre-existing conditions. It is also possible that some of the observed imaging findings can be seen in asymptomatic individuals, and thus, establishing a direct cause and effect link is not possible given the retrospective study design and beyond the scope of this paper.

The overall prevalence of Olympic athletes in our study being evaluated for shoulder pain (5%) is certainly lower than prior reported values in overhead throwing athletes [7, 8]. This however, is likely a reflection of the diversity of Olympic sports as well as the selection bias geared toward a group of patients in which MRI imaging was indicated. We did not include other modalities that are routinely used to evaluate shoulder pain, which include ultrasound, radiograph, and computed tomography (CT). This means we likely have an underestimation of the overall athletes evaluated for shoulder pain. Thus, this study is a modality specific analysis rather than a study of overall injury prevalence.

In regards to our image interpretation, using one observer limited our ability to study intra-and inter-observer variability. It has also been shown that a higher MRI imaging matrix, stronger field strength, and intra-articular contrast (arthrography) increase the sensitivity of diagnosing labral tear [39–41]. In our study, both 3 T and 1.5 T MRI scanners were used and no arthrograms were performed. Thus, it is possible that our study lacked optimal sensitivity for labral tear detection. Furthermore, we do not know if any of these elite athletes underwent surgery for their shoulder pathology and their outcomes.

## Conclusions

Our study demonstrates a high rate of bursal, rotator cuff, and labral pathology in Olympic athletes receiving MRIs at the 2016 Rio de Janeiro Summer Olympic Games. These findings imply that some degree of glenohumeral instability and impingement is occurring, likely due to fatigue and overuse of the dynamic stabilizers. Due to the diversity of Olympic sporting events, we lack sufficient numbers to draw any further sport-specific conclusions to the injuries exhibited here. More conclusive patterns may emerge by a combined analysis with future Olympic competitions. Since the cumulative effect of chronic injury and overuse syndromes in the elite athlete is an atypically higher rate of osteoarthritis [42], ultimately, it is our interest to continue understanding problems that affect those who have made such great sacrifices to represent their countries in international competition.

## Abbreviations

AC: Acromial Clavicular
CT: Computed Tomography
IOC: International Olympic Commission
IRB: Institution Review Board
MRI: Magnetic Resonance Imaging
PACS: Picture archiving communications system
PD: Proton Density
RIS: Radiological Information System
SLAP: Superior Labrum Anterior Posterior
